# Supplementary material for: Mycobacterium tuberculosis requires SufT for Fe-S cluster maturation, metabolism, and survival in vivo
Source: PLoS Pathog. 2022 Apr 15;18(4):e1010475. doi: 10.1371/journal.ppat.1010475 (PMC9045647; doi:10.1371/journal.ppat.1010475)
Supplement: S1 Table — (DOCX) [file ppat.1010475.s001.docx]

**S1 Table**. Details of primers used in this study.

| **No** | **Oligo Name** | **5'<-----Sequence----->3'** |
| --- | --- | --- |
| 3 | FP sufR RT | GCGCCGTATCGACGCCATCC |
| 4 | RP sufR RT | GCTTTGCTGAGCGCCGTTGC |
| 5 | FP Rv1461 RT | GCGACATCTCCGCGAAGAAG |
| 6 | RP Rv1461 RT | AATCGATGCCATCGAGGTTGG |
| 7 | FP Rv1462 RT | CGACGAGATCTGGCGGTTC |
| 8 | RP Rv1462 RT | ACGGTCTGGGTGTATACG |
| 9 | FP Rv1463 RT | ACCACGAGATCCCGATCCTG |
| 10 | RP Rv1463 RT | CGTAGGACAGCGTCGACTTG |
| 11 | FP Rv1464 RT | GTGGAAACCCGTTGGCG |
| 12 | RP Rv1464 RT | TTGGACGCGGTCAGGAACTC |
| 13 | FP Rv1465 RT | ACCAGGGTCACCGACGTTTC |
| 14 | RP Rv1465 RT | ACGCGTTGTCCGATTACCTG |
| 15 | FP Rv1466 RT | TGGTCTACGGCTTGGACGTG |
| 16 | RP Rv1466 RT | CGCGACTGATCCTCGATGAC |
| 17 | ahpC_FP-RT | TTTGGCCGAAAGACTTCACG |
| 18 | ahpC-RP_RT | GACATCAAGCGCGAACTCAG |
| 19 | SodA-FP | CAGACCTGGACTGGGACTAC |
| 20 | SodA-RP | TTCAGCAAGATCGCTGAGTG |
| 21 | katG_FP_RT | CACCCACCCATTACAGAAAC |
| 22 | katG_RP_RT | GGTGCAGTACCTTCAGATTG |
| 23 | IscS_FP_RT_MD | GACGGACTCGAGGAAAACAG |
| 24 | IscS_RP_RT_MD | AAAGTGAAGTGCGCGTTACC |
| 25 | 16s rRNA_FP_RT | TTGACGGTAGGTGGAGAAGA |
| 26 | 16s rRNA_FP_RT | CGCAAGGCTAAAACTCAAAGG |
| 27 | Rv1466_CRSPRiOligo_1 | GGGAGCACGTCCAAGCCGTAGACC |
| 28 | Rv1466_CRSPRiOligo_2 | AAACGGTCTACGGCTTGGACGTGC |
| 29 | Rv1466_CRSPRiOligo_3 | GGGAGCGGGCACGCCGCCGACGTG |
| 30 | Rv1466_CRSPRiOligo_4 | AAACCACGTCGGCGGCGTGCCCGC |
| 31 | FP_SufT_BamH1_MPFC_AT | TACAGGATCCATGAGCGAAACCAGCGCACCG |
| 32 | FP_SufT_EcoR1_MPFC_AT | ACGTGAATTCGAAGCCTTCGAGGAGGTTAC |
| 33 | RP_SufT_Hindlll_MPFC_AT | ACTGAAGCTTACGCGCCGGTTCAGACGGTG |
| 34 | FP_ACN_HINDlll_MPFC_AT | AGTCAAGCTTCGGGCGAACCAAATCCCTGG |
| 35 | RP_ACN_HINDlll_MPFC_AT | ACGTAAGCTTCGCTCGGTCAGCCTGACTTC |
| 36 | FP_SufR_BamHl_MPFC_AT | AGTCGGATCCCTCAATGTGACCAGCACAAC |
| 37 | RP_SufR_Hindlll_MPFC_AT | AGTCAAGCTTTGTCATCGGGACGCTCCTTC |
| 38 | Rv1464 FP_MPFC_Hindlll_AT | AGCTAAGCTTCCAACCGGAGCCTGACATGA |
| 39 | Rv1464 RP_MPFC_BamH1_AT | ACGTAAGCTTGACGCAACGTCACGCTCTTC |
| 40 | Rv1465 FP_MPFC_BamH1_AT | AGCTGGATCCCTTTGGAAGAGCGTGACGTT |
| 41 | Rv1465 RP_MPFC_Hindlll_AT | ACGTAAGCTTGTCGGCGAGCAATTCCTCAG |
| 42 | SufT_SDM C62-A F | CACGTCGGCGGCGGCACCGCTGACCGATGTCAT |
| 43 | SufT_SDM C62-A R | ATGACATCGGTCAGCGGTGCCGCCGCCGACGTG |
| 44 | FP_AT_Rv1466_Nde1 | ACTGCATATGAGCGAAACCAGCGCACCGGCTGAG |
| 45 | RP_AT_Rv1466_Xho1 | ATCGCTCGAGTTGCCGGATTCTCCGCCCTCAGTTC |
| 46 | FACN_Nde1_AT | ACGTCATATGGTGACTAGCAAATCTGTG |
| 47 | RACN_Xho1_AT | AGTCCTCGAGTCAGCCTGACTTCAGTATGT |
