# Supplementary figures and images for: Mycobacterium tuberculosis requires SufT for Fe-S cluster maturation, metabolism, and survival in vivo
Source: PLoS Pathog. 2022 Apr 15;18(4):e1010475. doi: 10.1371/journal.ppat.1010475 (PMC9045647; doi:10.1371/journal.ppat.1010475)

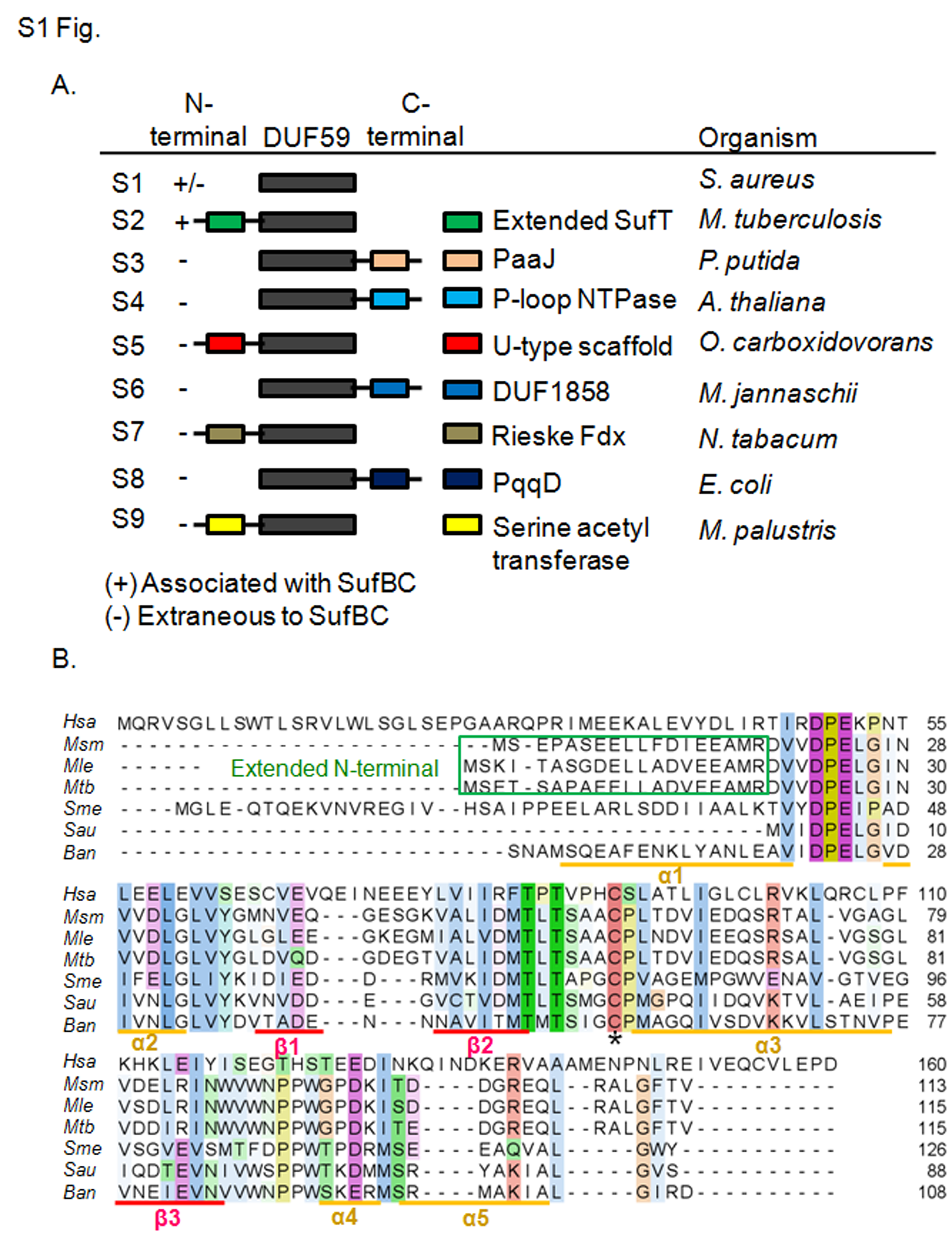

Supplement: S1 Fig — (A) Nine modular structures of DUF59 containing proteins, referred to as S1 to S9. Sign (+) and (-) indicate corresponding SufT proteins that are within four ORFs of sufBC in the genome (associated) or encoded at distance of more than four ORFs in the genome (extraneous). N- and C-terminal motifs are indicated with different colors, along with their homologous functional role and host organism. The Mtb SufT (Rv1466) is a representative member of the S2 structure. (B) Alignment of the diverse amino acid sequences of DUF59 domains from the H. sapiens, M. smegmatis, M. leprae, M. tuberculosis, S. meliloti, S. aureus, and B. anthracis showing conserved putative site residues (DPE-X26–31-T-X2/3-C). The green box indicates extended N-terminal sequence exclusively found in Mycobacterium sp., yellow and red color indicates the locations of five α-helices and three β-strands, respectively. A highly conserved and putative hyper reactive cysteine (C62) is star (*) marked. (TIF) [file ppat.1010475.s003.tif]

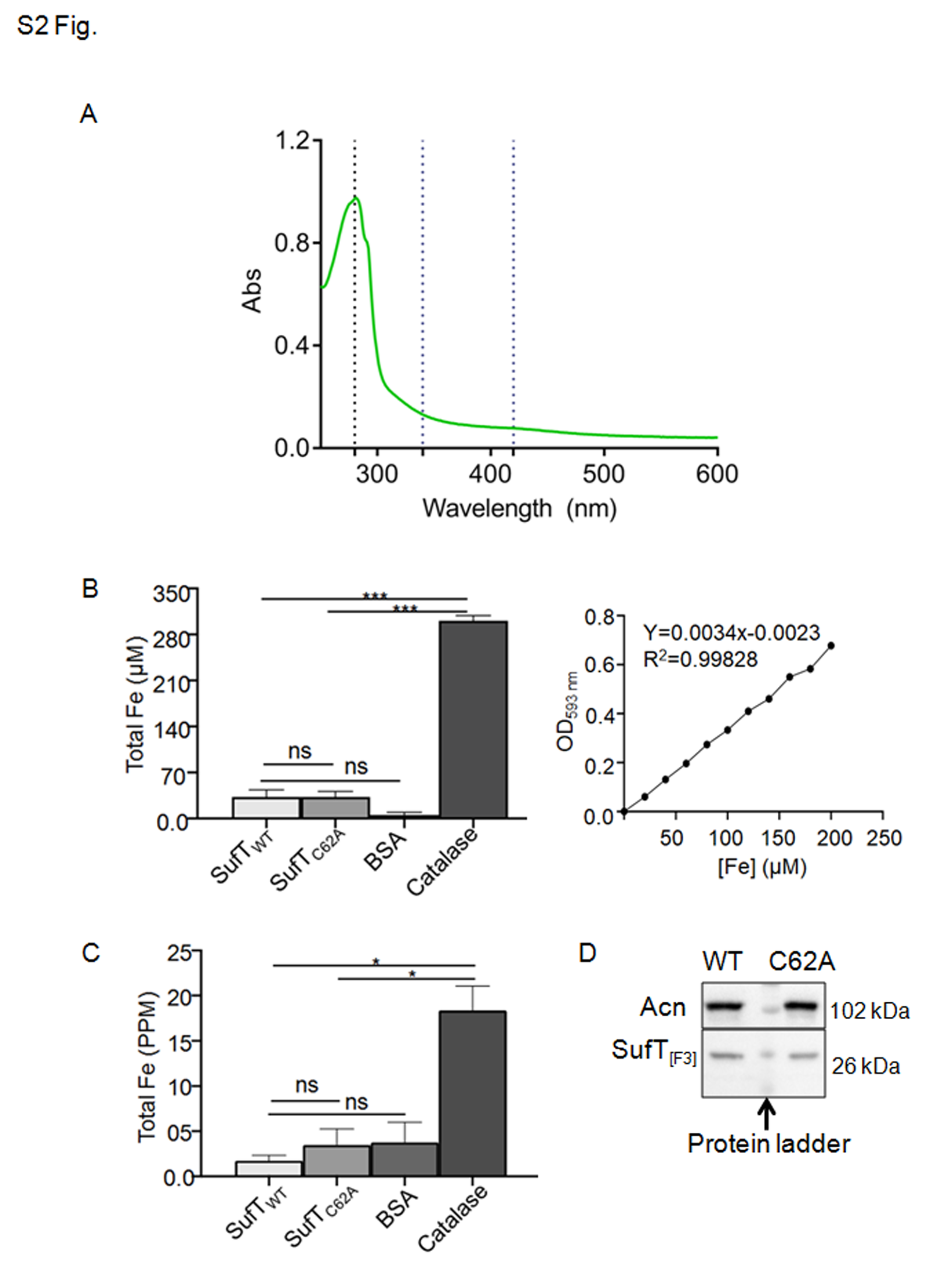

Supplement: S2 Fig — (A) Purified SufT was subjected to UV–visible spectroscopy. Absence of characteristic peak at 340 nm or 420 nm indicates that SufT is unlikely to be a Fe-S cluster containing protein. 100 μM of purified SufTWT and SufTC62A proteins were saturated with Fe2+ in presence of FeSO4 in vitro and total Fe was measured by B) biochemical assay and (C) atomic absorption spectrophotometer. Bovine serum albumin (BSA) and catalase were used as negative and positive control, respectively. (D) Western blot of SufTWT[F3] and SufTC62A[F3] expressed in host Msm for M-PFC experiments. Aconitase (Acn) is used as a loading control. Experiment was repeated three times and a representative image is shown. (TIF) [file ppat.1010475.s004.tif]

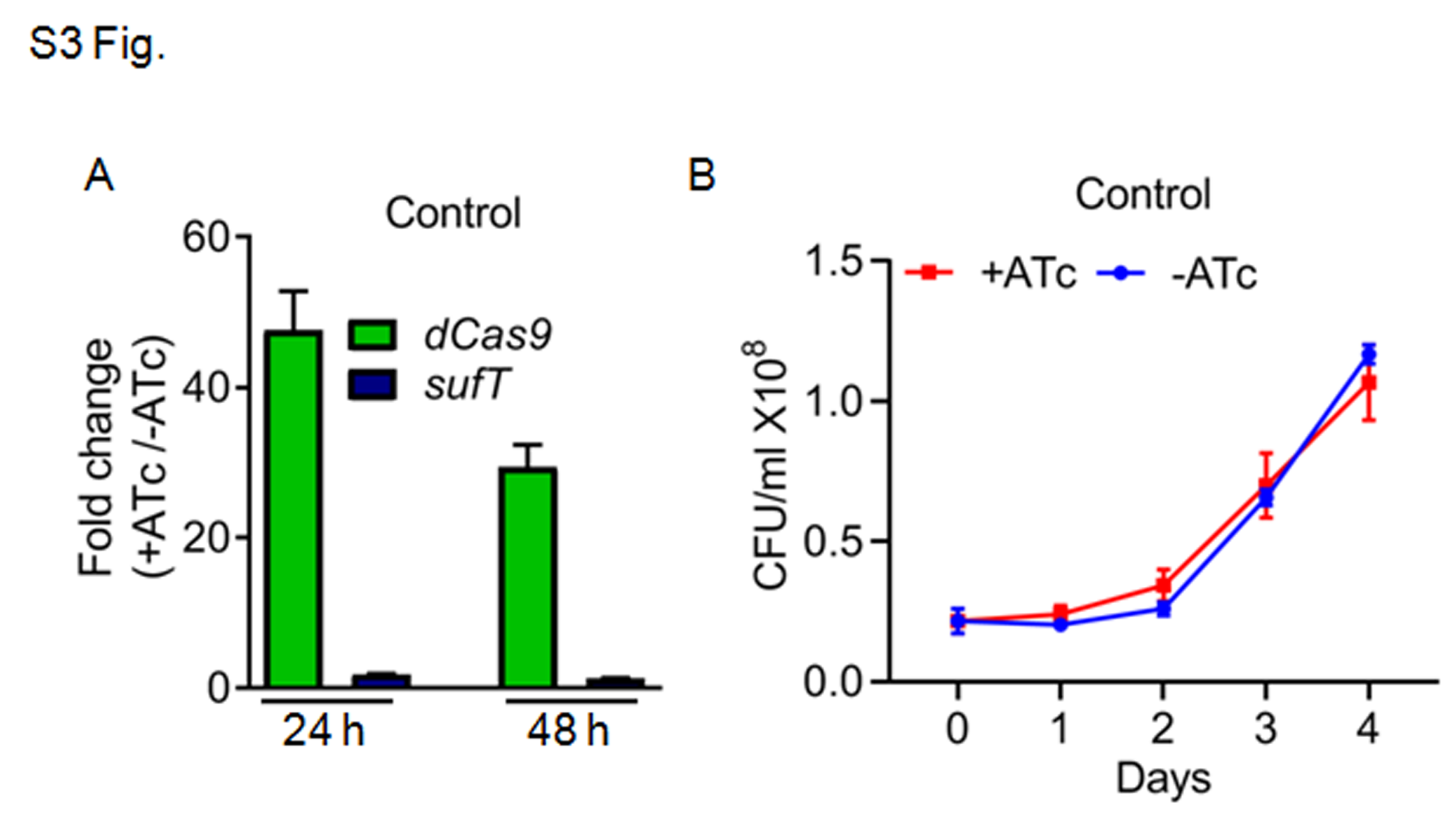

Supplement: S3 Fig — (A) The Mtb strain expressing pRH2502/pRH2521 vectors as vector control (control) was exposed to ATc (200 ng/mL) for 24 and 48 h and the expression of sufT and dCas9 was monitored by RT-qPCR. Experiment was performed in triplicate. (B) Growth curve in CFU/mL of control strain with and without ATc (200 ng/mL). (TIF) [file ppat.1010475.s005.tif]

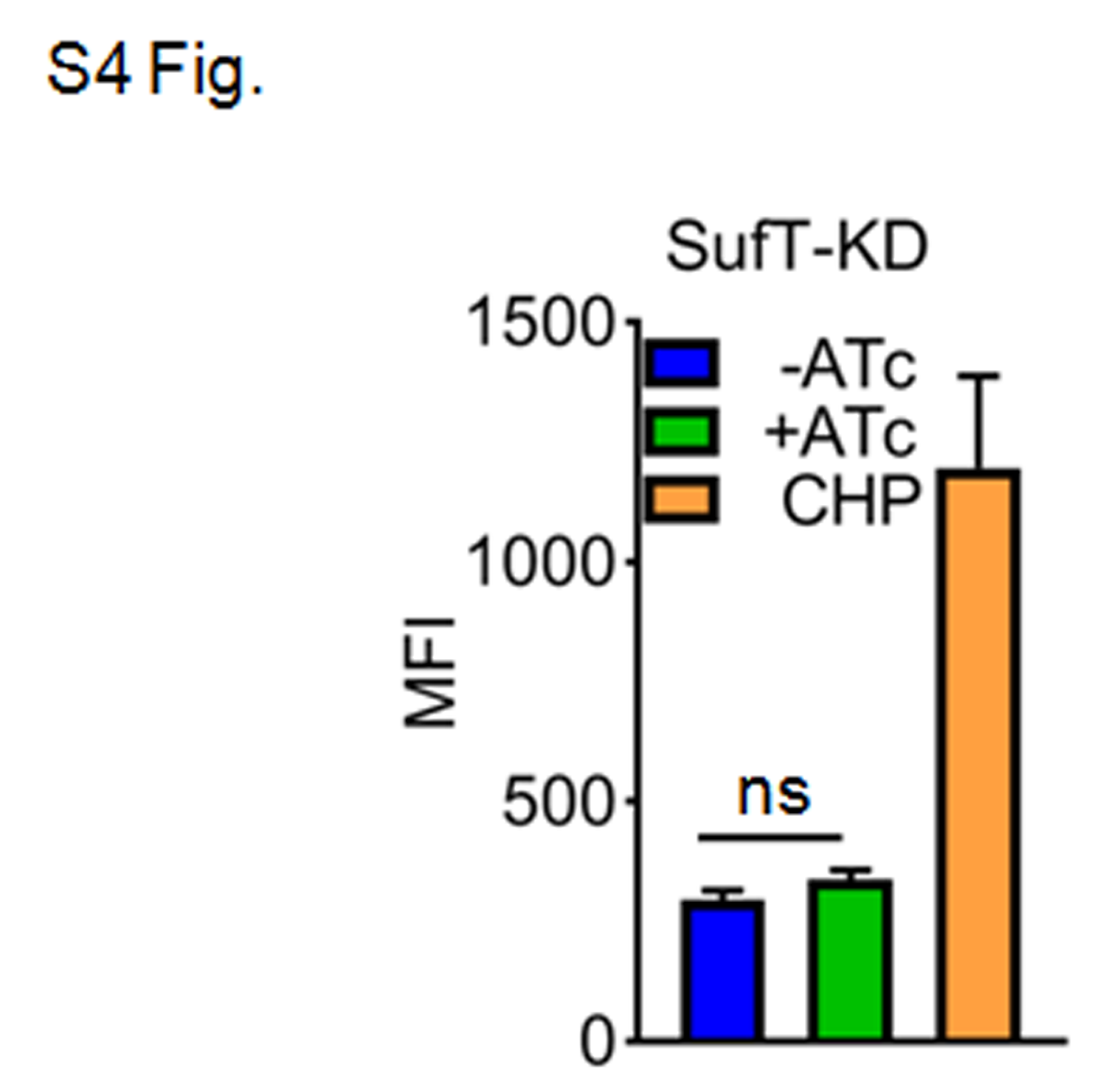

Supplement: S4 Fig — Endogenous ROS was measured in the SufT-KD strain after 72 h of treatment with and without ATc (200 ng/mL) by using CellRox Deep Red dye staining. MFI indicates median fluorescence intensity. Cumene hydroperoxide (CHP; 5 mM) treatment for 15 min was used as positive control. Experiment was performed in triplicate with two independent experiments. ns: non-significant based on the Student’s t-test. (TIF) [file ppat.1010475.s006.tif]

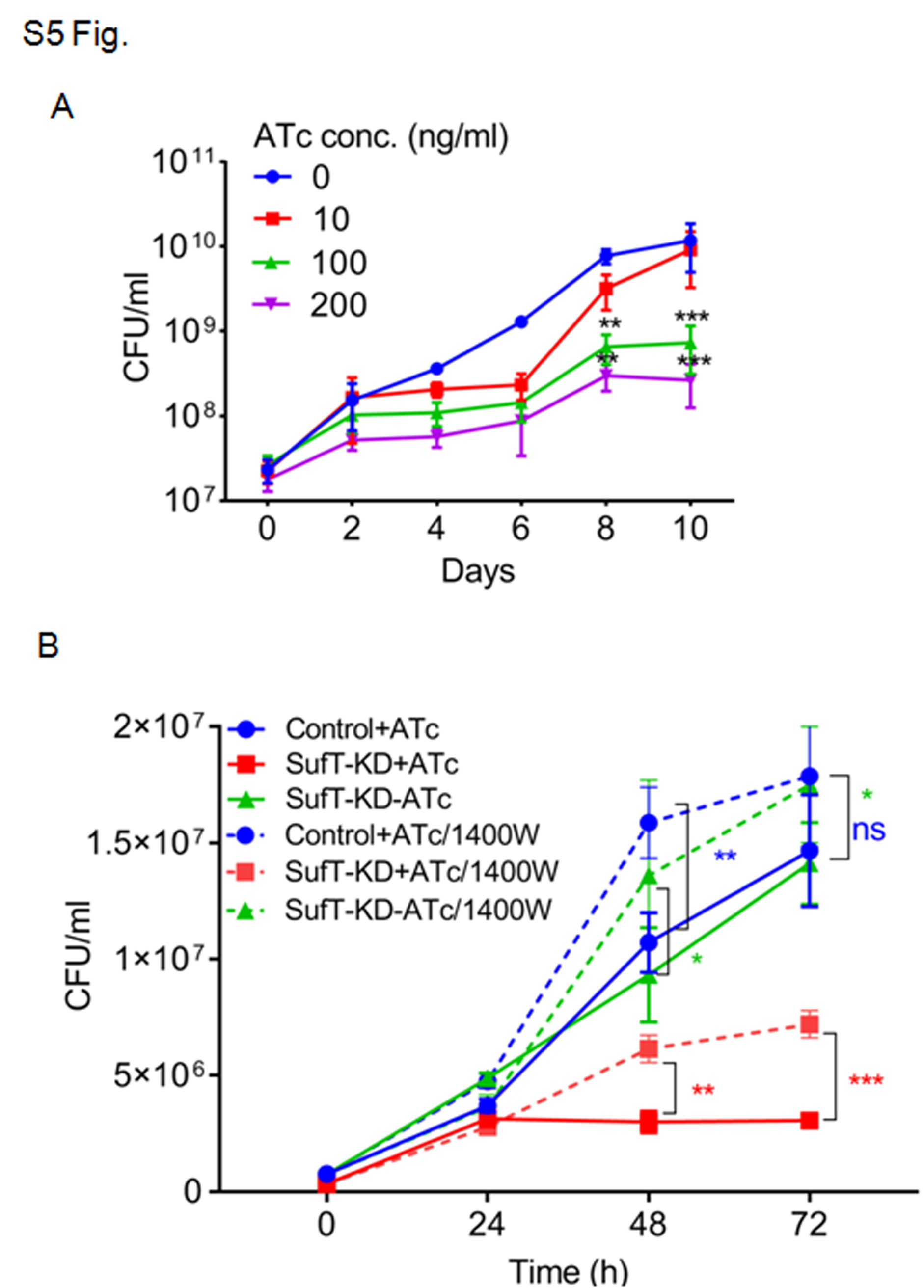

Supplement: S5 Fig — (A) CFU analysis of the SufT-KD strain at the indicated concentrations of ATc. Experiment was performed in triplicate with two independent experiments. Student’s t-test was applied to measure significance (p**≤0.01 and p***≤0.001). Defective survival of the SufT-KD strain in RAW264.7 is partly dependent on iNOS. (B) iNOS inhibitor partially rescued growth defect of the SufT-KD in RAW264.7. RAW264.7 cells were infected with control+ATc, SufT-KD+ATc and SufT-KD-ATc at MOI 1:2, and further incubated with 25 μM of iNOS inhibitor 1400W (dotted lines) or without 1400W (solid lines). CFU was performed at the indicated time points. Two biological experiments were performed, and the student’s T test was applied to calculate p value (p*≤0.05, p**≤0.01 and p***≤0.001). (TIF) [file ppat.1010475.s007.tif]
