# Supplementary material for: Mycobacterium tuberculosis requires SufT for Fe-S cluster maturation, metabolism, and survival in vivo
Source: PLoS Pathog. 2022 Apr 15;18(4):e1010475. doi: 10.1371/journal.ppat.1010475 (PMC9045647; doi:10.1371/journal.ppat.1010475)
Supplement: Attachment — Submitted filename: Response letter PPATHOGENS March 19h.docx [file ppat.1010475.s008.docx]

**PPATHOGENS-D-21-02388	19^th^ March 2022**

Dear Dr. Boshoff,

We thank the reviewers’ for responding enthusiastically to our manuscript "*Mycobacterium tuberculosis* SufT integrates Fe-S cluster maturation with core intermediary metabolism for survival *in vivo*". We also thank them for the providing thoughtful comments, which we have addressed in the revised manuscript. Below we provide point-by-point responses to the reviewer comments (yellow highlight). We also refer to specific line numbers in the revised manuscript for rapid editor cross-checking.

**Part II – Major Issues: Key Experiments Required for Acceptance**

**Reviewer #1**:

**No major issues**

We thank the reviewer to summarizing the work nicely and providing excellent feedback.

**Reviewer #2**: -

**The section "SufT depletion results in widespread metabolic realignment in Mtb" should be thoroughly reworded, and the claims should be toned down. Only correlations are reported here, not evidence of direct functional links. Similarly, the claim in the title of the manuscript should be mitigated. Metabolism is modified in the SufT-KD strain; the extent to which this is directly related to SofT activity is a complex question that would require far more experiments to be fully addressed.**

We thank the reviewer for this comment. As per the reviewer’s suggestion, we have reworded the metabolic realignment section and toned down the claims to reflect that depletion of SufT results in the modification of central metabolism. We also changed the title of the manuscript. The new title of the manuscript is “*Mycobacterium tuberculosis* requires SufT for Fe-S cluster maturation, metabolism, and survival *in vivo.”* We also indicated that the precise mechanism by which SufT influences metabolism needs future experimentation (page no. 15 and line number 338-339 and 449-450).

**- Fig. 4 would be more convincing if Fe-S clusters would be measured in Acn and SufR in the WT and sufT-KD strains.**

We thank the reviewer for raising the possibility of measuring Fe-S clusters on Acn and SufR in the WT and SufT-KD strains. However, these experiments are technically challenging even with non BSL3 class organisms. The 57Fe Mössbauer spectroscopy is the only non-invasive technique available for detecting Fe-S clusters in vivo (1). However, this procedure requires artificial overexpression of the Fe-S cluster proteins and a large volume of cells to detect signals. Overexpression of Fe-S cluster proteins at levels exceeding the amount required could either overwhelm Suf-driven Fe-S cluster biogenesis maturation/biogenesis or results in the accumulation of apo-form of Acn/SufR. Further, we need to perform paraformaldehyde fixation of Mtb cells before subjecting them to Mössbauer spectroscopy. This is likely to create an oxidation artifact. Therefore, we believe that Mössbauer spectroscopy is not appropriate for quantifying Fe-S clusters on proteins in Mtb. Other techniques are invasive (55Fe labelling and immunoprecipitation (2) and could only provide incorporation of Fe (not Fe-S clusters) on the proteins.

To test whether the depletion of Mtb SufT leads to altered activity of Fe-S cluster-dependent proteins, we quantified Acn enzymatic and SufR repressor activities in mycobacterial whole cell lysates. The [4Fe-4S] cluster of Acn is essential for the conversion of citrate into isocitrate, and Acn enzymatic activity is consistently taken as a measure of impaired Fe-S cluster biogenesis and maturation in diverse organisms including Mtb (3,4). Similarly, we have recently reported that [4Fe-4S] cluster of SufR is essential for the repression of the suf operon, and depression of the suf operon is indicative of loss of SufR 4Fe-4S cluster (5). Both of these assays show diminished activities of Acn and SufR in the SufT-KD strains compared to wt Mtb. We hope that the reviewer would agree that these findings, together with the data showing protein: protein interaction between SufT and SufR/Acn, support the role of SufT in Fe-S cluster maturation.

**- In Fig. 6 and 7, an important parameter to consider is the number/fraction of live bacteria in the sufT-KD population after 3 days of treatment with Atc.**

As suggested by the reviewer, we included CFU data of the SufT-KD strain after treatment with various concentrations of ATc. Consistent with the OD_600_nm data, depletion of SufT reduces the growth of *Mtb* as compared to the control strain (Fig1 [Fig S5A in the revised manuscript]). After three days of treatment with 200 ng/ml ATc, we observed 5-7-fold lesser CFU in the SufT-KD strain as compared to untreated cells (Fig. 2) .

,

**Fig 1. SufT depletion slows down growth of *Mtb*.** SufT-KD strain was treated with different concentration of ATc (0, 10, 100 and 200 ng/ml) and viable counts were determined at the indicated time points. Student’s t test was performed to calculate *p* value (*p***≤0.01 and *p****≤0.001).

**In 6A,B, to what extent if the reduced OCR and ECAR not due to reduced viability?**

We apologize for not describing the methodology clearly in the manuscript. For OCR and ECAR measurements, we have taken an equal number of cells (2x10^6^ cells/well) for Atc-treated and untreated cultures. Briefly, the SufT-KD cells were cultured and treated with 200 ng/ml of ATc for 72 h. Under these conditions, the viable counts for ATc-treated cells were 3.1x 10^7^/ml, whereas the counts were 1.5 x10^8^/ml for control cells (ATc-untreated). We diluted the cultures and adhered an equal number of viable cells (2x10^6^/ well) to the XF cell culture plates for OCR and ECAR measurements. Therefore, the data shown in Fig 6A and 6B are from an equal number of cells in both cases. The duration of the experiment was 80 min and OCR and ECAR measurements were done in the absence of ATc, ruling out any influence of ATc on the SufT-KD viability during the measurement period.

**Same in 7C, what is the fraction of live bacteria in the control (0 H2O2)?**

As explained earlier, all the strains (control, SufT-KD -Atc , and SufT-KD + Atc) were diluted, and an equal number of cells 2x10^7^ (input 0 hrs) were exposed to H_2_O_2_/NO. As shown in figure 2, CFU data of input bacteria at 0 h indicate comparable CFUs for the strains used in the experiment.

**Fig 2**. **CFU analysis to determine input bacterial numbers**. Control+ATc, SufT-KD-ATc, and SufT-KD+ATc were grown for 72 h, cultures were diluted to OD 600 nm of 0.15 and CFU was determined. CFUs of (A) undiluted (72 h post ATc treatment) and (B) diluted (input 0h; OD 600 nm of 0.15) cultures were shown.

- **Claims lines 406-408 would be better supported by experiments using KO cells or inhibitors of NOS/ROS synthesis.**

This is an excellent suggestion, and we appreciate the reviewer’s recommendation. As suggested by the reviewer, we assessed the survival phenotype of the SufT-KD strain in response to iNOS inhibitor 1400W (6). As shown in figure 3 (S5B Fig in the revised manuscript]), treatment of RAW264.7 macrophages with 1400W partially reversed the growth defect exhibited by the SufT-KD strain. Data suggest that the growth inhibition of the SufT-KD strain in macrophages is partly due to host-generated nitric oxide. Furthermore, these findings indicate that attenuated survival and persistence of the SufT-KD strain in mice could (at least in part) be dependent upon host pressures such as reactive nitrogen intermediates.

**Fig 3**. **Defective survival of the SufT-KD strain in RAW264.7 is partly dependent on iNOS.** RAW264.7 cells were infected with Control+ATc, SufT-KD+ATc, and SufT-KD-ATc at MOI 1:2, and further incubated with 25 μM of iNOS inhibitor 1400W (dotted lines) or without 1400W (solid lines). CFU was performed at the indicated time points. Two biological experiments were performed, and the student’s T test was applied to calculate *p* value (**p*≤0.05, *p***≤0.01 and *p****≤0.001).

**Reviewer #3: N/A**

**Part III – Minor Issues: Editorial and Data Presentation Modifications**
Please use this section for editorial suggestions as well as relatively minor modifications of existing data that would enhance clarity.

 **Reviewer #1:**

**Many of the quite broad phenotypes observed for SufT are not surprising and would be expected when disrupting Fe-S biosynthesis (derailed energetics, TCA imbalances). SufT being an essential protein complicates a rigorous and conclusive analysis of a depletion phenotype in mice. Although the authors do what they can to address this issue by using an inducible knockdown system, it is still difficult to know if the amount of doxycycline used in the mice leads to depletion to an extent that just arrests bacterial growth (as it does in vitro) rather than reveal a host-related phenotype. The paper is written clearly and Figures are also very clear.**

We thank the reviewer for raising the issue of host-related phenotype. We now provide macrophage data showing that inhibition of iNOS partially rescued the defective growth phenotype exhibited by the SufT-KD in macrophages (Fig. 3.[S5B Fig. in the revised manuscript]). We acknowledge that a comprehensive understanding of the contribution of host immune pressures in modulating the survival and persistence phenotype of the SufT-KD requires future experimentation.

**Other comments:**

Line 217: “(We next) asked if SufS directly associates with the proteins requiring Fe-S clusters…” I think this should read SufT.

The typographical error is corrected in the revised manuscript (Line no 208 in revised main manuscript).

**Figure 1B: The side chains are difficult to see and the amino acid numbering is unclear:**

We have replaced the figure for better clarity (Fig 1 in main manuscript).

**There is no C62 in the Mtb SufT sequence. If the numbering is following the *B. anthracis* numbering, including that sequence in the sequence alignment would help clarify.**

The sequence alignment shows residue cysteine (C ) at 62 position, which is aligned with the corresponding C90 present in the DUF59 protein from *Homo sapiens* (S1B Fig of the revised manuscript). We have also included sequence of *B. anthracis* in the alignment figure of the revised manuscript (S1B Fig of the revised manuscript).

**Also, do the other structures have Fe bound? Did the Mtb structure? That might explain the different distances of the highlighted residues.**

We confirm that none of the structures have Fe bound to them, including *Mtb*. Please see references indicating the absence of Fe in these structures (7,8).

**Reviewer #2**: -

**Fig. S2C (lines 169-172), the authors conclude that SufT does not contain Fe, catalase being used as a control (Fe-containing protein). This is not clear. It loks like there is Fe in SufT and that iron concentrations in SufT and catalase are similar. Please clarify.**

We apologize for the confusion and thank the reviewer for pointing this out. The discrepancy is because of significant variations among the SufT samples using this technique. Further, we have not subtracted the iron present in the blank (buffer only) sample after passing through PD10 column from the analysis. We repeated this experiment using multiple replicates, subtracted the buffer only values, and replotted the graph. As shown in Fig 4 (S2C Fig in the revised figure), iron levels were significantly higher in catalase than SufT/SufTC62A/BSA. These findings are consistent with the biochemical estimation of iron using ferene-based biochemical assay (S2B Fig. in the revised manuscript).

**Fig 4**. **SufT lacks bound Fe**. Purified SufT_WT_ and SufT_C62A_ proteins (100 μM) of were saturated with Fe^2+^ in presence of FeSO^­^_4_ *in vitro* and total Fe was measured by atomic absorption spectrophotometer. Bovine serum albumin (BSA) and catalase were used as negative and positive control, respectively. Two biological experiment were performed and the student’s t test was applied to calculate *p* value (*p**≤0.05).

**- Replace SufS by SufT, line 217**

We have corrected this typographical error in the revised manuscript (line no. 208 in revised main manuscript).

- The conclusion, lines 250-252, should better be positioned within the next section.

We have repositioned the conclusion in the next section. (Shifted to line no 242-244 in revised main manuscript)

**- Line 343, Asp is not shown in the figure.**

We have corrected this error in the revised manuscript (Line no. 330).

**- In Fig. 5, why were S-containing amino acids not included? This should be discussed.**

In our estimations, we only included targeted measurements of SAM, SAH and cystathionine as representative of sulfur amino acids (and the sulfur cycle), for the following reasons. With respect to cysteine, *Mtb* cells are generally known to maintain very low amounts of free cys (fluxing it to other 'end point' sulfur metabolites) (9,10), and more importantly (2) we did not include cys measurements because cys is extremely easily oxidized post-preparation (11). Furthermore, extraction preparation requires that the conditions for extraction keep cys protonated, e.g., in an acidic environment (but this precludes long term storage due to steady degradation), and the samples need to be analyzed immediately after extraction. To a lesser extent, this is also true for methionine. Hence, we decided to focus on SAH and cystathionine, which are stable, reliable to measure, and collectively  reliable indicators of overall sulfur amino acid cycles and amounts in almost every system studied. Cystathionine is the precursor to cys , and SAM (and thereby SAH) are the primary metabolic products of methionine, so together can be used to infer changes in cys (or cys-dependent metabolites), or methionine. We add clarifying lines to modify our statements to softer conclusions in the results (**line no 337-338**) to indicate this point.

**Fig. 8A, why is the WT strain not included?**

In our experiments, the WT strain (Vector control) and the SufT-KD strain without ATc treatment exhibited comparable growth phenotype in *in vitro* stress (Fig7C and 7D) experiments and inside macrophages (Fig 7E and 7F in the revised manuscript). Earlier published work also confirmed that the growth phenotype of WT strain (H37Rv) in the presence or absence of doxycycline (DOX) remained identical (12). On this basis, we only included SufT-KD strain without DOX as a control in our animal experiments.

**Reviewer #3**:

This manuscript needs to be significantly edited as its a very hard read and the findings of the manuscript are really lost in all the details. The abstract needs to be completely rewritten and all the acronyms replaced so that the paper is attractive to readers and the key points are emphasised.

We thank the reviewer for this comment. We have significantly edited the manuscript. Briefly, the title of the manuscript is revised. We have also rewritten the abstract and edited some parts of the results section for better clarity.

**The in silico and other protein work whilst interesting needs to be significantly edited down and perhaps some of he details could be removed and put in thesupplementary information. What is now shown about their biogenesis from this work needs to be clearly emphasised as its rather buried in places.**

As suggested by the reviewer, we have moved a part of in silico results in the supplementary information (S1B Fig).

**The metabolite measurements are interesting however this doesnt tell you anything about metabolic flux as inferred from the discussion. In order to do this the authors would have to perform some labelling.**

We thank the reviewer for this comment. The reviewer is correct in that only a pulse-label of a metabolite, and the estimation of label incorporation in the metabolic products can fully indicate flux, since the steady-state amounts cannot separate production from consumption. However, when observed over entire pathways, or complete sets of metabolites, they do conclusively indicate the overall metabolic state in the cell, and in many cases correlate with overall flux. However, to be correct in our description, in the text we now rephrase our conclusions to state that "these results show that these SufT-KD cells accumulate TCA cycle intermediates.

**References**:

1.	Khoroshilova N, Popescu C, Münck E, Beinert H, Kiley PJ. Iron-sulfur cluster disassembly in the FNR protein of Escherichia coli by O2: [4Fe-4S] to [2Fe-2S] conversion with loss of biological activity. Proc Natl Acad Sci U S A. 1997;94(12):6087–92.

2.	Kispal G, Csere P, Prohl C, Lill R. The mitochondrial proteins Atm1p and Nfs1p are essential for biogenesis of cytosolic Fe/S proteins. EMBO J. 1999;18(14):3981–9.

3.	Rybniker J, Pojer F, Marienhagen J, Kolly GS, Chen JM, Van Gumpel E, et al. The cysteine desulfurase IscS of Mycobacterium tuberculosis is involved in iron-sulfur cluster biogenesis and oxidative stress defence. Biochem J. 2014 May 1;459(3):467–78.

4.	Mashruwala AA, Bhatt S, Poudel S, Boyd ES, Boyd JM. The DUF59 Containing Protein SufT Is Involved in the Maturation of Iron-Sulfur (FeS) Proteins during Conditions of High FeS Cofactor Demand in Staphylococcus aureus. PLoS Genet. 2016;12(8):1–39.

5.	Anand K, Tripathi A, Shukla K, Malhotra N, Jamithireddy AK, Jha RK, et al. Mycobacterium tuberculosis SufR responds to nitric oxide via its 4Fe–4S cluster and regulates Fe–S cluster biogenesis for persistence in mice. Redox Biol. 2021;46:102062.

6.	Braverman J, Stanley SA. Nitric Oxide Modulates Macrophage Responses to Mycobacterium tuberculosis Infection through Activation of HIF-1α and Repression of NF-κB. J Immunol. 2017 Sep 1;199(5):1805–16.

7.	Mashruwala AA, Boyd JM. Investigating the role(s) of SufT and the domain of unknown function 59 (DUF59) in the maturation of iron–sulfur proteins. Curr Genet. 2018;64(1):9–16.

8.	Peti W, Herrmann T, Zagnitko O, Grzechnik SK, Wüthrich K. NMR structure of the conserved hypothetical protein TM0979 from Thermotoga maritima. Proteins Struct Funct Genet. 2005;59(2):387–90.

9.	Wheeler PR, Coldham NG, Keating L, Gordon S V., Wooff EE, Parish T, et al. Functional demonstration of reverse transsulfuration in the Mycobacterium tuberculosis complex reveals that methionine is the preferred sulfur source for pathogenic mycobacteria. J Biol Chem. 2005;280(9):8069–78.

10.	Gould TA, Van De Langemheen H, Muñoz-Elías EJ, McKinney JD, Sacchettini JC. Dual role of isocitrate lyase 1 in the glyoxylate and methylcitrate cycles in Mycobacterium tuberculosis. Mol Microbiol. 2006;61(4):940–7.

11.	Schöneich C. Mechanisms of Protein Damage Induced by Cysteine Thiyl Radical Formation. Chem Res Toxicol. 2008 Jun;21(6):1175–9.

12.	Gandotra S, Schnappinger D, Monteleone M, Hillen W, Ehrt S. In vivo gene silencing identifies the Mycobacterium tuberculosis proteasome as essential for the bacteria to persist in mice. Nat Med. 2007 Dec 2;13(12):1515–20.
